# Treatment response in children and adolescents with anorexia nervosa: a naturalistic, case–control study

**DOI:** 10.1007/s40519-022-01425-3

**Published:** 2022-06-15

**Authors:** Jacopo Pruccoli, Ilaria Pettenuzzo, Antonia Parmeggiani

**Affiliations:** 1 IRCCS Istituto delle Scienze Neurologiche di Bologna, Centro Regionale per i Disturbi della Nutrizione e dell’Alimentazione in Età Evolutiva, UO Neuropsichiatria dell’Età Pediatrica, Bologna, Italy; 2 Dipartimento di Scienze Mediche e Chirurgiche (DIMEC), Università di Bologna, Bologna, Italy

**Keywords:** Treatment-resistant, Developmental age, Adolescents, Anorexia nervosa, Eating disorders, Third-level center for feeding and eating disorders, Response to treatment

## Abstract

**Purpose:**

Although a few recent articles describe adults with treatment-resistant anorexia nervosa (TR-AN), no study addresses the specific features of subjects not responding to treatment in the developmental age. This study reports on the clinical and psychopathological variables that distinguish children and adolescents who did not respond to treatment (here “TR-AN”) from good-outcome controls, in a multidisciplinary hospital treatment setting.

**Methods:**

Naturalistic, case–control study conducted on individuals showing lack of response to treatment and good-outcome controls. TR-AN was defined as two or more incomplete admissions and no complete admissions, consistently with studies in adults. Good-outcome was defined as complete first admission, availability for follow-up visit after 6 months, and maintaining at follow-up a %BMI > 70% in the absence of binging or purging in the preceding 3 months. Psychopathological (Eating Disorders Inventory-3 EDI-3; Beck Depression Inventory-II), clinical, and treatment variables at admission were compared. Significant differences in the univariate analyses were included in an exploratory binary logistic regression.

**Results:**

Seventy-six patients (30 TR-AN, 46 good-outcome AN controls) were enrolled (mean age 14.9 ± 1.9 years, *F* = 94.7%). TR-AN individuals had a higher age at admission and higher EDI-3 Eating Disorder Risk (EDRC) scores, were treated less frequently with a nasogastric tube (NGT), and achieved a lower BMI improvement at discharge than good-outcome controls. A predictive model for TR-AN status was found (*X*^2^ = 19.116; Nagelkerke-*R*^2^ = 0.478, *p* < 0.001), and age at admission (OR = 0.460, *p* = 0.019), EDI-3 EDRC (OR = 0.938, *p* = 0.043), and NGT (OR = 8.003, *p* = 0.019) were associated with a TR-AN status.

**Conclusions:**

This is the first report on the psychopathological and clinical characteristics of children and adolescents not responding to treatment. These patients showed higher age and eating disorder scores, and were less frequently fed with NGT than controls. Despite the multiple incomplete admissions of our subjects, the short included follow-up limits the possibility for direct comparisons with adult samples of treatment-resistant patients. Thus, the specific features of children and adolescents with TR-AN should be assessed in longitudinal studies.

**Level of evidence:**

III, Observational, case–control study.

## Introduction

Anorexia nervosa (AN) is a complex eating disorder (ED) that typically begins in late childhood and adolescence. AN encompasses many clinical, psychological, and environmental variables [1].

While some patients fully recover after a single episode, more than 20% of individuals with AN exhibit a fluctuating pattern of weight gain followed by relapse, or experience a chronic course over many years [2]. The lack of substantial clinical response is an open challenge for clinicians. It is well documented that the earlier treatments start, the better the outcomes will be [2].

Many different treatment settings are available to individuals with AN, ranging from outpatient to inpatient programs according to the gravity of the illness [3]. Many studies have established that when an inpatient setting is required, overall remission rates tend to be lower [1]. Individuals with AN, achieving poor inpatient treatment outcomes, show high rates of dropout and readmission, which often lead to treatment-resistant AN [4, 5].

While a clear definition of “treatment-resistant” AN could help guide doctors’ early clinical decisions, the literature lacks a univocal definition for individuals with AN who are resistant to treatment. Different categorizations for this condition have been proposed, such as “treatment-resistant” AN, “severe” AN, and “severe and enduring” AN [6].

The severity of AN is defined in the Diagnostic and Statistical Manual of Mental Disorders—fifth edition DSM-5 by body mass index (BMI), ranging from mild (> 17) to extreme (< 15) in adults, and using BMI percentile in children and adolescents [1]. Studies and guidelines addressing the young population have identified a cutoff of < 70% of BMI for severe malnutrition, in addition to evidence of clinical signs and symptoms, such as bilateral pitting edema, inability to stand, or apparent dehydration [7, 8]. Other studies have identified severe AN as chronic AN, as defined by the duration of core symptoms for more than 7–10 years [6, 9, 10].

On the other hand, the term “treatment-resistant” AN (TR-AN) has been used in the literature for individuals with a personal history of multiple care accesses (to inpatient or outpatient eating disorder services) and several dropouts [5, 11, 12]. These patients usually leave the program under the targeted weight, which leads them to multiple re-admissions to inpatient programs [13–15]. TR-AN summarizes the concept of unsuccessful treatment attempts [16]. However, a precise number of incomplete treatments or re-admissions that would define TR-AN have not been established throughout the different studies [17–19].

Moreover, a relevant question remains about identifying the potential predictors of TR-AN among diagnosis, age of onset, duration of disease, psychopathology, and comorbidities [11, 15, 20]. Recently, a study by Smith and colleagues has specifically addressed the clinical characteristics of adult subjects with TR-AN [15]. To the best of our knowledge, no study so far has analyzed the clinical characteristics and predictors of the outcome of TR-AN in a cohort of young patients.

The primary aim of this study is to describe a sample of children and adolescents with “TR-AN” in the naturalistic context of a multidisciplinary hospital intervention. This sample is compared to good-outcome controls from the same setting and period. Our secondary aim is to retrospectively describe clinical variables that may help recognize young patients who will later develop “TR-AN”. Early identification of clinical variables associated with the development of a TR-AN status, if progressively confirmed by further studies, could help clinicians and researchers spot out those patients who could present a scarce response to treatment, and develop specific interventions to address treatment-resistant subjects.

Given the exploratory nature of the research, as well as the lack of previous comparisons in the developmental age, no pre-specified hypothesis was made for this study.

## Methods

### Study design and participants

This is an observational, naturalistic, case–control study. The study was conducted in the context of an observational survey investigating the use of psychopharmacological treatments in a third-level center for children and adolescents with feeding and eating disorders (FED), and was approved by the local ethical committee (code NPI-DAPSIFA2020). The evidence here reported focuses on the included hospitalized subjects with AN, representing the largely preponderant group of patients referring to the Center and undergoing a standardized protocol of assessments during the treatment. Strengthening the Reporting of Observational Studies in Epidemiology (STROBE) guidelines were followed during the planning and conducting of the study [21].

The study was conducted in December 2021, retrospectively considering all the patients assessed at the center between 1 January 2016 and 31 December 2020, and with at least one hospitalization for ED in the same center. Hospitalization was defined as inpatient or day-hospital treatment. This day-hospital treatment program for patients with ED is comparably structured and as intensive as inpatient treatment. The hospital program adopted in our center has been previously described [22] and provides a multidisciplinary psychological, nutritional, and psychopharmacological intervention.

Inclusion criteria were: (a) diagnosis of AN according to the DSM-5 criteria [1]; (b) hospitalization in the center where the study was conducted; (c) age up to 18 years; and (d) acquisition of informed consent. The exclusion criterion was the lack of necessary clinical documentation. Included patients were then screened for a possible condition of “TR-AN” (case group) or “good-outcome AN” (control group), according to a definition matching the one used by Smith and Woodside in conceptualizing treatment-resistant AN in an adult sample [15]. Patients were considered “to have a good outcome” if they completed their first admission, were available for the follow-up visit after 6 months, and maintained a %BMI > 70% at follow-up in the absence of binging or purging in the preceding 3 months.

The use of %BMI was preferred to standard BMI, as indicated by the report “Junior MARSIPAN: Management of Really Sick Patients under 18 with Anorexia Nervosa” to assess underweight in children and adolescents [23]. Percentage BMI was calculated using standardized growth charts, as (BMI/median BMI for age and gender × 100) [23]. In this study, a %BMI > 70% threshold was adopted, consistently with the same “Junior MARSIPAN” document, which defines young patients at the highest risk as those with less than 70% median BMI for age and gender, for whom hospital admission is likely [23]. The World Health Organization BMI-for-age growth charts for girls and boys were used as reference values in this study [24].

Patients were considered to be TR-AN if they had two or more incomplete admissions and no complete admissions in the study period. As in [15], patients with multiple incomplete admissions and admissions without known outcomes were not included. Patients not falling in any of these two categories (TR-AN or good-outcome AN) were excluded from the study. Given the retrospective nature of the study, missing data were not replaceable.

### Assessment methods

Diagnoses of AN were performed by clinicians trained in the field of FED. All patients were subjected to a psychological, nutritional, and psychiatric inpatient treatment regime. Demographic and clinical data were obtained for all patients. BMI was recorded at hospital admission and discharge. Duration of illness before hospitalization and duration of hospitalization were also considered. The psychopathological assessment of the included patients was performed using three standardized measures:

- The Eating Disorders Inventory-3 (EDI-3), which is a self-assessment questionnaire routinely used in the diagnosis of ED symptoms shown to be clinically relevant in individuals with ED [25]. The questionnaire consists of 91 items organized into 12 scales, three scales of which are specific to FED (Drive for Thinness—DT; Bulimia—B; Body Dissatisfaction—BD) and nine scales of which assess general psychological symptoms (Low Self-Esteem—LSE; Personal Alienation—PA; Interpersonal Insecurity—II; Interpersonal Alienation—IA; Interoceptive Deficits—ID; Emotional Dysregulation—ED; Perfectionism—P; Asceticism—A; Maturity Fears—MF), highly relevant to FED. The scale has reliability coefficients ranging from 0.83 and 0.90, and the various composite scales have test–retest reliability coefficients between 0.84 and 0.87. The Italian version of EDI-3 has very good documented day test–retest reliability, a good discriminating validity, and cross-informant agreement [26].
- The Beck Depression Inventory-II (BDI-II), which is a widely used psychological assessment for the severity of depression [27]. The test consists of 21 items, investigating the severity of depressive symptoms: sadness, pessimism, past failure, loss of pleasure, guilty feelings, punishment feelings, self-dislike, self-criticalness, suicidal ideation or wishes, crying, agitation, loss of interest, indecisiveness, feelings of worthlessness, loss of energy, change in sleeping pattern, irritability, change in appetite, concentration difficulty, tiredness or fatigue, and loss of interest in sex. The subject taking the test chooses the score (from 0 to 3) to best describe their symptoms in the last 2 weeks. The total test score ranges from 0 to 63, with higher scores documenting higher levels of depressive symptomatology [27].
- The Self Administered Psychiatric Scales for Children and Adolescents (SAFA), which is a validated psychometric tool used to assess psychiatric comorbidities in children and adolescents with ED [28, 29]. The SAFA consists of a series of tests organized into six scales, with each scale divided into different subscales. The assessments target a spectrum of psychopathological symptoms: anxiety-related (SAFA-A), depression-related (SAFA-D), somatic-related (SAFA-S), obsessive–compulsive (SAFA-O), psychogenic eating disorders-related (SAFA-P), and phobic (SAFA-F) symptoms. A recent study has reported on the Cronbach’s test showing α values of 0.84 for answers to SAFA anxiety questions, and 0.77 for SAFA depression [30]. The full test series has been administered to all patients considered for our study, to systematically assess potential comorbid psychiatric symptoms. The results were not reported here to avoid over-specification and potential biases due to multiple comparisons.

In this study, for the measurement of ED-related psychopathology, we used the EDI-3 composite scores at admission for ED risk (EDRC) and global psychological maladjustment (GMPC) [25], while depressive symptoms were assessed using a Beck Depression Inventory (BDI-II), which considers the total score at admission [27]. Treatment variables (medications, use of NGT) were also taken into account.

### Statistical analysis

Descriptive statistics for demographic and clinical variables included means and standard deviations, or absolute and percentage frequencies. TR-AN and good-outcome AN groups were then compared using Student t tests for continuous variables when appropriate (Mann–Whitney test for non-parametric distributions), and Chi-square test for categorical variables (Fisher’s exact test when needed). Bonferroni correction for multiple comparisons was applied. Significant variables in the univariate analyses were selected for inclusion in an exploratory binary logistic regression to assess the different contributions of the single variables to the prediction of a TR-AN status. Given the aims of the study and following the study on adults previously mentioned [15], outcome variables (BMI difference, discharge BMI, and length of hospital stay) of the two groups were compared, but not included in this last analysis. The significance level was set at 0.05, and all tests were two-tailed. All statistical analyses were conducted using SPSS, version 26 for Windows.

## Results

### Patients included in the study

**Fig. 1:**
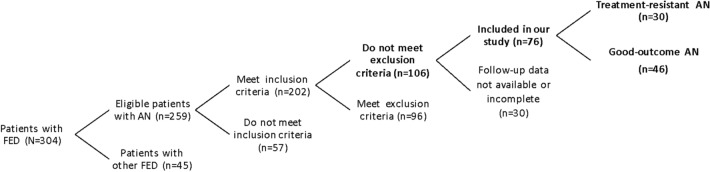
Flowchart describing the selection process of the patients included in the study. *FED* Feed and Eating Disorders, *AN* anorexia nervosa

The clinical documentation of 259 patients with AN referring to our third-level Regional Center for FED in developmental age, during the selected period, was thoroughly reviewed. Two hundred and two patients met the inclusion criteria. Of these patients, 106 were retained after applying the exclusion criteria. Of these patients, 76 were retained in the final analysis: 30 patients had multiple incomplete admissions and no complete admissions (TR-AN); 46 completed their first admission, continued to be well, and were available for the follow-up visit after 6 months (good-outcome AN). The flowchart of the enrollment process is reported in Fig. 1.

### Study sample and comparisons between the two groups

Seventy-six patients (*F* = 72, 94.7%) were enrolled in the study, with a mean age of 14.9 (± 1.9) years. The mean BMI at admission was 14.3 (± 1.7) kg/m^2^, while the mean BMI at discharge was 16.0 (± 1.6) kg/m^2^. At the 6-month follow-up, the mean BMI was 17.3 (± 2.3) kg/m^2^ for the available individuals (good-outcome subjects). The comparison of the main demographic, clinical, and treatment variables between the two groups are reported in Table 1. Individuals with “TR-AN”, when compared with those with a good outcome, had a significantly higher age at admission (*p* = 0.004) and a higher EDI-3 EDRC score (*p* = 0.004), were treated less frequently with an NGT (0.046), and obtained a lower BMI improvement at discharge (*p* = 0.003). No statistically significant differences were noted as regards the psychopharmacological treatment, AN subtypes, and psychopathological comorbidities, nor in the duration of untreated illness or hospitalization.

**Table 1:** Comparison of the assessed variables between individuals with “TR-AN” and AN with a good outcome

Variables	TR-AN	Good-outcome AN	Significance
Demographic variables (1)			
Sex	*F* = 28 (93.3%) *M* = 2 (6.7%)	*F* = 44 (95.7%) *M* = 2 (4.3%)	*X*^2^ = 0.196, *p* = 0.645
Age (years)	15.7 (± 2.0) (range 10–17)	14.4 (± 1.7) (range 10–17)	***U* = 712.000, *p* = 0.004**
DUI (months)	18.0 (± 14.2)	11.7 (± 7.4)	*U* = 833.000, *p* = 0.071
Admission BMI	14.7 (± 1.8)	14.0 (± 1.7)	*U* = 705.000, *p* = 0.107
Diagnoses (1)			
AN subtype	ANR = 25 (83.3%) AN-BP = 5 (16.7%)	ANR = 45 (97.8%) ANBP = 1 (2.2%)	*X*^2^ = 5.245, *p* = 0.022
MDD	2 (6.7%)	2 (4.4%)	*X*^2^ = 0.196, *p* = 0.658
OCD	6 (20.0%)	6 (13.0%)	X2 = 0.661, *p* = 0.416
Psychopathology (1)			
BDI-II	31.3 (± 9.0)	25.6 (± 13.3)	*U* = 175.000, *p* = 0.235
EDI-3 EDRC	78.4 (± 17.9)	65.6 (± 23.2)	***U* = 493.000, *p* = 0.004**
EDI-3 GMPC	79.1 (± 24.4)	76.1 (± 30.3)	*U* = 425.000, *p* = 0.270
Psychopharmacology (1)			
SSRI	28 (93.3%)	41 (89.1%)	*X*^2^ = 0.384, *p* = 0.536
AAP	24 (80.0%)	36 (78.3%)	*X*^2^ = 0.000, *p* = 1.000
Mood stabilizers	3 (10.0%)	1 (2.2%)	*X*^2^ = 2.230, *p* = 0.294
Nutritional interventions			
NGT	10 (33.3%)	23 (50.0%)	***X***^**2**^** = 3.994, *p* = 0.046**
Outcome variables (1)			
LOS (days)	111.5 (± 72.0)	126.8 (± 98.3)	*U* = 346.500, *p* = 0.697
Discharge BMI	15.8 (± 2.0)	16.0 (± 1.3)	*U* = 550.000, *p* = 1.000
BMI difference	1.2 (± 1.0)	2.0 (± 1.0)	***U* = 320.500, *p* = 0.003**

(1) Bonferroni corrected level of significance adjusted for a series of 3 (*p* = 0.050/3 = 0.017)

*AAP* atypical antipsychotics, *AN* anorexia nervosa, *ANR* anorexia nervosa restrictive subtype, *ANBP* anorexia nervosa binge-purging subtype, *BDI-II* Beck depression inventory, second edition, *BMI* body mass index, *DUI* duration of untreated illness, *EDI-3* eating disorders inventory, third edition, *EDRC* eating disorder risk composite, *GMPC* global psychological maladjustment composite, *LOS* length of hospital stay, *MDD* major depressive disorder, *NGT* nasogastric tube feeding, *OCD* obsessive–compulsive disorder; *SSRI* selective serotonin reuptake inhibitors, *TR-AN* treatment-resistant anorexia nervosa

### Logistic regression

Age at admission, EDI-3 EDRC score, and the use of NGT were included as independent variables in the logistic regression, with the group status (TR-AN vs good-outcome AN) as a dependent variable.

The logistic regression model is reported in Table 2. The resulting model was found to be statistically significant (*X*^2^ = 19.116; Nagelkerke-*R*^2^ = 0.478, *p* < 0.001), with no evidence of multicollinearity. The examination within the model of the independent variables documented that age at admission, EDI-3 EDRC, and NGT remained statistically significant predictors of a treatment-resistant status. When compared with good-outcome AN subjects, individuals with “TR-AN” had a significantly higher age and a higher EDI-3 EDRC score at admission, and were treated less frequently with an NGT.

**Table 2:** Logistic regression model assessing patients’ characteristics associated with “TR-AN” or good-outcome AN status

Independent variables	OR	95% CI	*P* value
Omnibus likelihood ratio			< 0.001
Age at admission	0.460	(− 1.421 to − 0.130)	0.019
EDI-3 EDRC	0.938	(− 0.125 to − 0.002)	0.043
NGT	8.003	(0.341 to 3.819)	0.019

*AN* anorexia nervosa, *EDI-3 EDRC* Eating Disorders Inventory, *third edition* Eating Disorder Risk Composite, *NGT* nasogastric tube feeding, *TR-AN* treatment-resistant anorexia nervosa

## Discussion

The primary aim of our study was to describe a sample of children and adolescents with “TR-AN”, in the naturalistic context of a multidisciplinary hospital intervention in a third-level Regional Center for FED in developmental age. This study also aimed to identify the variables that may predict a TR-AN status. To the best of our knowledge, this is the first study to directly address and describe a “TR-AN” in a population of children and adolescents.

A group of 30 children and adolescents with “TR-AN” was studied in this research; the sample size allows to expand on the results reported in adults while providing insights into the condition during the developmental age [15].

Smith and Woodside compared adult patients with AN, who completed their treatment and maintained weight after 1 year of follow-up, with patients with AN who had two or more incomplete admissions to specialist inpatient ED services and no complete admissions in the study period (the latter ones were categorized as TR-AN patients). We adopted the same criteria for the two groups in our study with the only difference that we considered as “good outcomes” the individuals who completed their first admission, continued to be well, and were available for the follow-up visit after 6 months.

While Smith and Woodside described the binge-purge subtype AN to be more difficult to treat in adults (62.2%), in line with prior studies [15, 15], in our sample, ANR represented the most frequent AN subtype (83.3%).

More severe ED psychopathology and depressive symptoms, together with a shorter duration of the first admission program, were identified as good predictors in the adult sample [15]. Relevantly, one-fifth (20.0%) of our “TR-AN” patients presented a comorbid OCD.

It is worth noting that several of our patients, regardless of evolution, received a psychopharmacological treatment (SSRI and AAP) (Table 1). While these drugs have no specific indication for the treatment of AN [31], they could help manage anxiety and AN depressive symptoms; furthermore, their known propensity to cause weight gain could be beneficial to underweight individuals. However, especially when considering developmental-age patients, clinicians should consider the potential exposure of TR-AN individuals to multiple pharmacological interventions and the risk that they develop treatment-related adverse side effects and reduced compliance.

The comparison between good-outcome AN and “TR-AN” patients documented a series of characteristics that could independently help recognize “TR-AN” in the early stage of the illness. Specifically, the “TR-AN” subjects in our sample were of a significantly higher age at admission, had higher EDI-3-EDRC scores, used less NGT, and showed a lower BMI difference between admission and discharge.

A major finding of this study is represented by the older age of “TR-AN” patients at admission when compared to the good-outcome AN group. Conflicting evidence exists on the relationship between age and outcome in AN. Mairs and Nicholls observed that outcomes in adolescents are better than in adults, and that the percentage of relapses is lower among adolescent individuals [32]. It has also been suggested that childhood-onset AN has poorer outcomes than adolescent-onset AN, due to children’s lesser comprehension of their disorder [33]. Our findings contrast, in part, with this suggestion, perhaps on account of the different methodology adopted in previous studies to define good and poor outcomes: outcomes were evaluated according to Morgan and Russel’s outcome score, which is based on weight and menstruation [34]. At the same time, in an adult cohort of AN patients, age at onset did not differ between TR-AN and good-outcome AN groups [15]. This may be because, in adulthood, age-related differences are less evident, since the developmental process is almost completed.

In our sample, EDI-3 Eating Disorder Risk Composite scored higher in the “TR-AN” group, highlighting a more severe core eating disorder psychopathology. EDRC includes a drive for thinness, bulimia, and body dissatisfaction subitems of the EDI-3 scale. This finding is in line with the available literature on adults, as the severity of ED beliefs and cognition (scored through the EDE-Q scale) predicted resistance to treatment in AN patients [15].

According to our study, a NGT was significantly less frequently used in the “TR-AN” group. Although the role of NGT for refeeding remains unclear, it is known that NGT may help facilitate weight restoration in medically unstable patients with inadequate intake. We reported that patients treated with early AAP and early NGT showed lower LOS than those treated with late AAP [22]. Other studies suggest that NGT feeding leads to complete remission or to a significantly longer mean relapse-free period [30], and consequently a better outcome for the disorder. The choice of not using NGT in most “TR-AN” patients of our sample may have been caused by a slightly higher BMI at admission for this group (although no statistically significant difference was observed). Alternatively, the more severe psychopathology and a lack of comprehension of the illness may have challenged adherence to medical treatment and may have consequently led to a difficult co-operation with the medical equipment, resulting in “TR-AN” patients’ refusal of NGT feeding.

According to our study, the three variables described above (age at admission, more severe EDI-3 Eating Disorder Risk Composite, and NGT usage) can independently help to identify AN patients with a high risk of treatment-resistant disorders. We suggest that these characteristics could be considered predictors of resistance to cure. Further research should investigate how to minimize unsuccessful treatment when individuals who could develop TR-AN are detected early.

In contrast with Smith and Woodside [15], depressive symptoms did not appear to be more severe in our “TR-AN” group. In our study, Beck Depressive Inventory total score did not differ between the two groups, nor did differences emerge as regards major depression disorders comorbidity. It is relevant to note that depressive symptoms are more challenging to recognize, if present, in children and adolescent AN cohorts, since young patients could report somatic complaints more easily and have a comorbid anxiety disorder [35–37]. Another study did not consider depressive symptoms at admission as relevant for differentiating between a good outcome and relapsing AN in adolescent patients: the authors suggested that scoring tests at admission could be unreliable due to the severity of illness (malnourishment, fatigue, separation from the home environment, and medical complications) [38]. We think that longitudinal studies addressing depressive symptoms in young individuals with “TR-AN” could provide significant insights into this subject.

One last point requires consideration, as binge-purging anorexia nervosa is known to have a higher risk of developing TR-AN [15, 15]. In our study, this subtype of AN did not appear to be related to a specific AN group, in contrast with Smith and Woodside’s results [15]. The different result could be due to a low number of ANBP subtypes in our study group (5/30 in the “TR-AN” group and 1/46 in the “good outcome-AN” group), causing a lack of statistically significant difference that Bonferroni correction controlled. To verify such a comparison, two comparable groups of AN subtypes (binge-purging and restrictive) should be investigated.

However, it is worth observing that no statistical differences emerged in the length of hospital stay between the two groups. These data seem to suggest that a patient’s early dropout does not lead to the development of TR-AN”, nor does a longer period of untreated illness help identify a worse outcome.

Our study, which is the first one in the literature to compare young patients with “TR-AN” and good-outcome AN, presents some limitations. First, the “TR-AN” subjects included in our research presented a relatively short duration of untreated illness (18.0 ± 14.2 months) compared to that of the adult reference sample (median: 6.84 years, interquartile range 2.62, 12.32). However, duration of untreated illness was not included in the criteria for TR-AN adults, and the age at evaluations (median 24.00 years, interquartile range 21.50, 30.00) greatly differed from the one reported in this study (15.7 ± 2.0 years) [15]. A significant concern in establishing direct comparisons with adult TR-AN arises from the short follow-up considered here (6 months). Despite the multiple incomplete hospital admission presented by our “TR-AN” sample, clinicians should carefully consider that young patients with AN may show clinical characteristics that may lead to final better outcomes, as recently reported [39]. Thus, the “TR-AN” group here included should be more cautiously considered as “not responding” to the administered treatment regime. Nonetheless, adolescents with acute AN may suffer a greater reduction in brain gray matter than adults, and complete long-term recovery from these alterations is not clear in these subjects, as documented by a recent meta-analysis [40]. Therefore, clinicians and researchers should consider the specific relevance of AN symptoms occurring during the developmental age, especially when, as in the current study, subjects are exposed to multiple hospitalizations and incomplete treatment regimes.

Moreover, the retrospective nature of the study did not allow us to verify the natural history of AN at defined checkpoints. Second, we use the Smith and Woodside definition for TR-AN which is very strict and may lead to identifying predictive variables for only a subgroup of patients with very severe AN. Thirdly, childhood and adolescence AN is quite different from adult AN; a comparison between the two groups is not easy, and many of our observations and suggestions should be further investigated in comparative age-related studies. Finally, although we verified that “TR-AN” predictors are independent in recognizing the worse AN outcome through the logistic regression model, dependent items, not included in our analysis, could have influenced the results. These limitations notwithstanding, the presence of a control group and the adoption of criteria matching those used in a recent study on adults made it possible to gain significant insights by comparing children and adolescents with TR-AN with adults with the same condition.

## Conclusions

This is the first study to document a sample of children and adolescents with “TR-AN” compared to good-outcome AN subjects in the context of a multidisciplinary treatment program for ED in a third-level Center for FED. When compared to good-outcome controls, individuals with “TR-AN” presented a higher age at admission, higher scores on ED-related psychopathology, and were less frequently treated with NGT. AN subtypes and depressive symptomatology did not differ between the two groups. These results may guide future, longitudinal studies, assessing prognostic factors related to treatment-resistant ED.

### What is already known on this subject?

The clinical and psychopathological characteristics of individuals with treatment-resistant anorexia nervosa (TR-AN) have been recently documented in a few studies. Research conducted in adult samples has reported that subjects with TR-AN may display greater eating disorder psychopathology and depressive symptoms at admission, as well as a higher frequency of binge-purging AN when compared to good-outcome individuals. The literature currently lacks studies conducted on children and adolescents showing lack of response to treatment (TR-AN).

### What does this study add?

When compared to good-outcome subjects, children and adolescents showing lack of response to treatment have a higher age and higher eating disorders psychopathology at admission, are treated less frequently with a nasogastric tube, and achieve a lower BMI improvement at discharge. This study reports for the first time the psychopathological and clinical characteristics of a group of children and adolescents with TR-AN, in a naturalistic multidisciplinary hospital setting.

## Data Availability

The data sets used and analyzed during the current study are available from the corresponding author on reasonable request.
